# Acute Kidney Injury: Biomarker-Guided Diagnosis and Management

**DOI:** 10.3390/medicina58030340

**Published:** 2022-02-23

**Authors:** Soo-Young Yoon, Jin-Sug Kim, Kyung-Hwan Jeong, Su-Kang Kim

**Affiliations:** 1 Department of Medicine, Graduate School, Kyung Hee University, Seoul 02453, Korea; lynnyoon41@gmail.com; 2 Division of Nephrology, Department of Internal Medicine, Kyung Hee University Medical Center, Seoul 02453, Korea; jinsuk0902@naver.com; 3 Department of Biomedical Laboratory Science, Catholic Kwandong University, Gangneung 25601, Korea

**Keywords:** acute kidney injury, biomarker, diagnosis, management, prediction, prognosis

## Abstract

Acute kidney injury (AKI) is a common clinical syndrome that is characterized by abnormal renal function and structure. The Kidney Disease: Improving Global Outcomes (KDIGO) Controversies Conference in 2019 reviewed the stages of AKI and the definitions of AKI-related terminologies, and discussed the advances in the last decade. Along with serum creatinine level and urine output, more accurate novel biomarkers for predicting AKI are being applied for the early detection of renal dysfunction. A literature search was conducted in PubMed, Scopus, Medline, and ClinicalTrials.gov using the terms AKI and biomarker, combined with diagnosis, management, or prognosis. Because of the large volume of data (160 articles) published between 2005 and 2022, representative literature was chosen. A number of studies have demonstrated that new biomarkers are more sensitive in detecting AKI in certain populations than serum creatinine and urine output according to the recommendations from the Acute Disease Quality Initiative Consensus Conference. To be specific, there is a persistently unresolved need for earlier detection of patients with AKI before AKI progresses to a need for renal replacement therapy. Biomarker-guided management may help to identify a high-risk group of patients in progression to severe AKI, and decide the initiation time to renal replacement therapy and optimal follow-up period. However, limitations such as biased data to certain studied populations and absence of cutoff values need to be solved for worldwide clinical use of biomarkers in the future. Here, we provide a comprehensive review of biomarker-based AKI diagnosis and management and highlight recent developments.

## 1. Introduction

Acute kidney injury (AKI) is a common condition that occurs in 5.0–7.5% of hospitalized patients and in 50–60% of critically ill patients [1,2,3]. The current criteria for diagnosing AKI are a sharp decrease in glomerular filtration rate (GFR), as represented by an acute increase in serum creatinine (SCr) levels or a decrease in urine output (UO) over a fixed period [4]. During the past few decades, an increasing number of studies have been conducted to standardize the definition and diagnosis, as well as to improve the understanding of AKI.

Biomarkers are being developed for anticipating AKI, and literature published between 2005 and 2022 was searched in PubMed, Scopus, Medline, and ClinicalTrials.gov using the terms AKI and biomarker, combined with diagnosis, management, or prognosis. Among 160 articles, selection of studies was restricted to randomized clinical trials, meta-analyses, or systematic reviews of adults investigating AKI, biomarkers, or renal replacement therapy (RRT) in the setting of critical illness related to sepsis or surgery. Unpublished studies were excluded. In retrieved data, several biomarkers have been suggested to diagnose AKI and evaluate the progression of AKI [5,6,7,8,9,10,11,12,13,14,15]. Owing to limited sensitivity and specificity, only a few grading models have been clinically validated, despite the need for a risk-stratification system for AKI [16].

Biomarker-based assessments of AKI severity or progression may help predict prognosis and set treatment directions for each individual patient. This review integrates recent data on a number of AKI biomarkers that are currently in use or being studied in different clinical situations.

## 2. Diagnosis of AKI

### 2.1. Definition and Diagnostic Criteria

#### 2.1.1. Definition of AKI and Types of Biomarkers

AKI is usually diagnosed when the SCr level has increased by >1.5 times the baseline value within the last 7 days or when the GFR has decreased by >25%. Biomarker measurements, kidney biopsy, and imaging evaluations may be crucial for classifying the cause, stage, and prognosis [17].

AKI and chronic kidney disease (CKD) are related disease entities. Although the duration of kidney disease is not a major factor in the current definition of AKI, it is associated with the prognosis of AKI [18]. The term “acute kidney disease” was recently suggested to describe prolonged AKI, defined as kidney injury persisting for >7 days but <3 months [19]. Recently, despite the definition of these terms, biomarker-related research is being conducted to compensate for the shortcomings of early diagnosis and prognostic evaluation of AKI.

**Figure 1:**
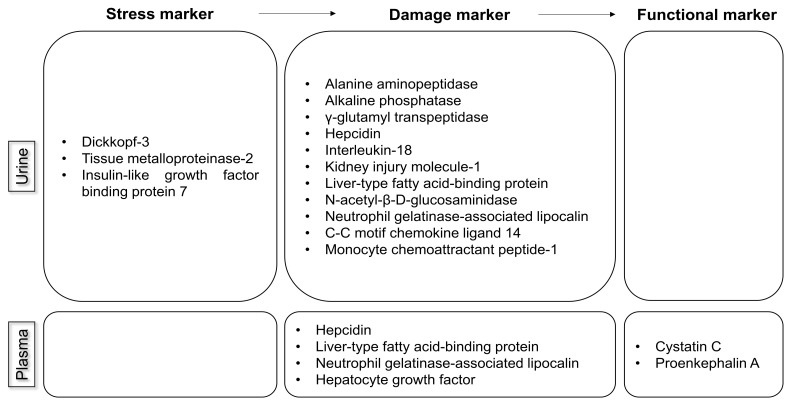
Three types of biomarkers of AKI from urine and plasma.

Three types of biomarkers exist based on the recommendations on AKI biomarkers from the Acute Disease Quality Initiative Consensus Conference (Figure 1). Stress markers reflect cell stress, which may resolve or become aggravated [7]. A damage marker indicates structural damage that may or may not result in a reduction in renal function [7]. Functional markers correlate with alterations in glomerular filtration [7]. Considering these biomarkers together can offer a precise approach beyond measuring the SCr level or UO alone, and may suggest the most accurate diagnostic and therapeutic methods.

#### 2.1.2. Biomarkers for Diagnosis

There is an unresolved need for earlier detection of AKI before its progression to kidney dysfunction requiring RRT. Biomarkers associated with AKI have been identified and clinically studied to contribute to the early diagnosis of the condition (Table 1). For instance, cystatin C (CysC) is a cysteine proteinase inhibitor released by nucleated cells, and the serum CysC level measured at various time points predicted AKI in some studies in patients undergoing cardiac surgery and in hospitalized patients [10,20]. Considering damage biomarkers together with functional biomarkers such as CysC and proenkephalin A can aid in accurately diagnosing AKI, differentiating pathophysiologic pathways, demonstrating AKI etiology, and grading AKI severity [7]. The urinary markers tissue metalloproteinase-2 (TIMP-2) and insulin-like growth factor binding protein 7 (IGFBP7), which are recently discovered inducers of G1 cell-cycle arrest and are key stress biomarkers of AKI, are considered superior to known damage biomarkers such as kidney injury molecule-1 (KIM-1) and neutrophil gelatinase-associated lipocalin (NGAL) [21]. Interleukin (IL)-18, a pro-inflammatory cytokine that induces the production of interferon gamma, is detected in urine after acute proximal tubular damage [10,22]. Urine KIM-1, a transmembrane glycoprotein, is a proven marker of AKI in adults [23]. NGAL is a key polypeptide found in blood and urine at the time of AKI development after ischemic or toxicity-induced damage in the kidney [24]. Besides the aforementioned biomarkers, various other biomarkers have been studied in specific populations based on their biological roles, despite their limitations, as summarized in Table 1 [7].

### 2.2. Risk Stratification for AKI Assessment and Prevention

#### 2.2.1. Causes and Risk Factors

A kidney health assessment requires detailed history taking, including current medications and any recent exposure to nephrotoxic agents, along with a precise physical examination and serology or urine tests for the appropriate categorization of AKI [27]. The volume status and clinical symptoms of congestive heart failure and other systemic infections should also be assessed in the initial evaluation [27]. Kidney biopsy is recommended to identify the intrinsic cause of AKI when it occurs with sudden deterioration of proteinuria or hematuria without known causes. The furosemide stress test (FST) has been introduced and standardized for assessing tubular integrity and nephron function without a kidney biopsy in patients with suspected early AKI; however, further trials are required to demonstrate its feasibility [28]. Prior to the application of biomarkers for AKI evaluation, efforts for finding causes of AKI and assessing the patient’s condition and underlying diseases should be carried out first.

#### 2.2.2. Risk-Stratification Models

AKI is difficult to predict because only a few causes are renal-specific and the condition can be easily stimulated by other systemic abnormalities [2]. Various studies have created prediction models based on whether the patient group is under an intensive care unit setting or not, whether the patient underwent cardiac or noncardiac surgery, and whether the model is a logistic regression or machine-learning model; however, the performance of these models has been inconsistent [29,30]. Additionally, the study showed that blood urea nitrogen levels, UO, age, and diabetes mellitus are also crucial predictors of AKI [31]. Moreover, clinical decision support (CDS) systems have been increasingly used to guide decision making in some in-hospital AKI cases [32].

However, current CDS systems have not shown a statistically significant effect on mortality and length of hospital stay [32]. Another limitation of AKI risk-stratification models is that the oversegmented groups of patients make them difficult to widely apply in the clinical setting. Nevertheless, stratification models in specific conditions keep developing. Several studies have suggested risk-stratification models to predict postoperative AKI before the patient undergoes cardiac or noncardiac surgery [33,34]. Since the ideal risk-classification model for risk of AKI is currently being studied in clinical practice, it is necessary to make an effort to analyze potential biomarkers which are available for clinical use, such as NGAL, and [TIMP-2] × [IGFBP7] as AKI risk factors. In future, sequential urine or plasma biomarker results might be conducted on those patients stratified as being at high risk of AKI development, through a machine-learning model.

#### 2.2.3. Biomarkers for AKI Risk Assessment, Prediction, and Prevention

Numerous serum and urinary biomarkers of AKI have been identified and proposed. Specifically, Dickkopf-3 (DKK3), a 38-kDa stress-induced glycoprotein derived from kidney tubular epithelial cells, is a urinary stress biomarker that has a potential role in the risk assessment and prediction of AKI [35]. Along with DKK3, other urinary biomarkers, such as TIMP-2/IGFBP7, IL-18, and KIM-1, have been demonstrated to be associated with both the prediction and diagnosis of AKI [9,10,12,25]. For instance, some biomarkers such as TIMP-2/IGFBP7 expanded the clinical impact for predicting AKI from intensive care units to emergency departments [25]. Therefore, various biomarkers can be implemented to precisely identify patients with AKI, diagnose AKI at an early stage, and perform risk stratification of patients who require dialysis or are at an increased risk of death [9].

## 3. Management of AKI

### 3.1. Conventional Management of AKI

#### 3.1.1. Hemodynamic Management

Two major factors of AKI in need of hemodynamic management are sepsis and surgery. The main causes leading to septic AKI are dysfunction in microcirculation, inflammation, decreased metabolism, and cell-cycle arrest [36]. Blood pressure may influence glomerular filtration and organ perfusion including the kidney directly in septic conditions [36]. Secondly, perioperative hemodynamic care may significantly reduce the prevalence of surgery-associated AKI; however, international hemodynamic optimization strategies or guidelines have not been developed [37].

When hypovolemia is suspected to be the cause of AKI, the fluid balance should first be restored to the normal level. Fluid administration in the case of severe tissue hypoperfusion appeared to result in less AKI compared to standard fluid therapy [38]. Some vasoactive drugs can induce renal perfusion through systemic vasoconstriction and elevated blood pressure [39]. An adequate dose of norepinephrine in a state of vasodilatory shock can reduce the risk of AKI [39,40]. Vasopressin is a common second-line treatment, in combination with norepinephrine, for increasing blood pressure and stabilizing hemodynamics. In a study comparing norepinephrine alone with the combination of norepinephrine and vasopressin, the combination group showed a trend toward a lower risk of AKI [41]. To ensure the appropriate treatment of AKI, it is essential to consider the patient’s characteristics and comorbidities when selecting or combining vasoactive drugs.

Most patients prescribed vasoactive drugs are critically ill patients, and they are at high risk of AKI. Since most of the biomarkers organized in Table 1 were studied on patients in the intensive care unit (ICU), these biomarkers might have the potential to detect the development of AKI prior to an SCr increase in patients at high risk of AKI. Effort has been gradually made in the introduction of biomarkers into clinical settings, but to date most of these biomarkers remain research tools and have not been incorporated into routine clinical practice. It is time to consider a process that allows promising biomarkers to overcome real-world barriers and improve clinical outcomes.

#### 3.1.2. Drug Stewardship and Use of Biomarkers

Nephrotoxicity induced by various therapeutic drugs is an important cause of AKI. The mechanisms of drug-induced nephrotoxicity vary among the different drug classes [42,43,44]. Therefore, the prescription of certain drugs should be cautiously reconsidered. The 2012 KDIGO guidelines recommend immediate cessation of potentially nephrotoxic agents, avoidance of radiocontrast exposure, and proper renal dose reduction in patients with renal impairment [45].

Biomarkers can also be used to predict the occurrence of AKI associated with the use of certain drugs. One medical center in the United States of America developed a CDS alert system to manage ICU patients exposed to nephrotoxic drugs [46]. Patients at high risk of drug-induced AKI can be identified through this alert, and be selected for novel biomarker testing. Biomarkers such as KIM-1, NGAL, and [TIMP-2] × [IGFBP7] can be used for the early identification of patients at high risk of developing AKI, thus allowing for timely, proper management [46,47]. Specifically, KIM-1 has been shown to be useful in the early detection of cisplatin- and amphotericin-related AKI [8,48]. Furthermore, NGAL levels have been shown to reflect cisplatin- and amphotericin-induced AKI 4.5 and 3 days earlier, respectively, than SCr levels [8,49].

Considering different biomarkers together may have clinical advantages. KIM-1 and NGAL levels have been shown to be significantly elevated in patients with vancomycin-associated AKI and to increase earlier than SCr levels [50]. The combination of nephrotoxin stewardship and novel biomarkers is expected to provide meaningful advances in the management of AKI.

### 3.2. RRT after Failure of Conventional Management

#### 3.2.1. Timing of RRT Initiation and Follow-Up after RRT

Approximately 5% of patients admitted to the intensive care unit because of AKI undergo RRT. Several randomized controlled trials have demonstrated that an earlier initiation of RRT does not give patients a survival benefit when compared with a “wait-and-see” or delayed strategy [51,52,53]. This management trend in the initiation of RRT makes it more challenging for clinicians to initiate RRT until clear evidence emerges because of a lack of clarity on optional conditions. As well as new biomarkers, some tests such as the FST have aided decisions on patient-centered RRT initiation with their growing clinical roles.

The FST has been standardized for assessing tubular integrity and nephron function in patients at a high risk of AKI development and RRT initiation, and previous studies have shown that the FST has better diagnostic performance than SCr levels in identifying early AKI cases [54].

Like the FST, the use of three sequential types of biomarkers can help categorize patients requiring RRT and determine the optimal timing of RRT initiation. A recent meta-analysis in critically ill patients with AKI demonstrated that there was no survival benefit to initiating RRT early [55]. Additionally, early initiation of RRT resulted in an increase in RRT-associated adverse events [55]. The conventional indications for the initiation of RRT in patients with AKI are severe or refractory hyperkalemia, uncorrectable metabolic acidosis, refractory volume overload, anuria, critical azotemia, and uremic complications (e.g., encephalopathy, pericarditis, and neuropathy) [56]. With early initiation of RRT in cases with clear and hard indications, patients with AKI could benefit from RRT.

Patients who require persistent RRT at the time of hospital discharge often undergo hemodialysis in outpatient dialysis clinics [57]. Identifying patients at a higher risk of developing CKD after an AKI episode is crucial. The risk factors for CKD after AKI include the severity, duration, and recurrence of AKI; time to recovery; advanced age; presence of diabetes, hypertension, congestive heart failure, or proteinuria; and a high Charlson comorbidity index [58,59,60]. After recovery from a critical illness and AKI, patients require thorough monitoring to achieve complete recovery to the baseline health condition.

#### 3.2.2. Biomarkers for Assessing AKI Progression and Reversal

Patients with complete AKI reversal within 48–72 h showed better clinical outcomes than those with persistent AKI; however, the definitions of persistent AKI differed among studies [7]. Thus, for standardization, the Acute Disease Quality Initiative meeting defined persistent AKI as renal injury that continues for >48 h and proposed applying biomarkers to assess patients at risk of AKI progression [7].

A number of novel diagnostic biomarkers are also related to both AKI severity and kidney recovery among the aforementioned biomarkers such as such as alanine aminopeptidase, alkaline phosphatase, γ-glutamyl transpeptidase, CysC, hepcidin, TIMP-2, IGFBP7, KIM-1, NGAL, and proenkephalin A in Table 1. These biomarkers are meaningful enough to confirm that they are AKI-related, but the other three biomarkers in Table 2, which have not yet been demonstrated for their role in diagnosis, have been sufficiently studied in the recovery and severity of AKI.

Representative biomarkers can facilitate the prediction of AKI reversal. In a large heterogeneous cohort study, urinary C-C motif chemokine ligand 14 levels were higher in critically ill patients with persistent AKI [13]. Higher urinary hepatocyte growth factor (HGF) levels were associated with disease severity, and the HGF level decreased to the baseline value in patients recovering from AKI [14]. A definite increase in the plasma monocyte chemoattractant peptide-1 level has been correlated with AKI progression and higher mortality after cardiac surgery [15]. Similar to biomarkers for AKI diagnosis and management, biomarkers for AKI progression and reversal are statistically more significant when they are combined with other biomarkers such as KIM-1 or NGAL [14]. The discovery of novel biomarkers as predictors of persistent AKI and renal nonrecovery may lead to therapeutic approaches for improving the prognosis of AKI (Table 2).

## 4. Limitations of Novel Biomarkers and Future Research Directions

The performance of novel biomarkers needs to be validated in certain situations with a known time of AKI occurrence, such as in patients undergoing cardiac surgery or coronary angiography, rather than in situations with an ambiguous timing of kidney injury, such as in patients with sepsis [6]. Additionally, the evidence is insufficient to recommend the practical use of new biomarkers for acute kidney disease staging. The integration of new biomarkers into routine clinical practice is limited to a few countries and cases, such as NGAL in Europe, liver-type fatty acid-binding protein in Japan, and the urinary biomarker [TIMP-2] × [IGFBP7] in the United States and Europe. For example, the cutoff levels of NGAL have not been standardized. The lack of NGAL cutoff levels with high sensitivity and specificity is a major limitation in real-world settings [61,62]. The variable cutoff values in published articles, risk of confounding by comorbidities, and high expenses are other barriers to overcome in future clinical studies.

Studies on approximately 20 representative biomarkers have proven that [TIMP-2] × [IGFBP7] and KIM-1 are potential biomarkers in clinical practice with roles in the diagnosis, prevention, and prognosis prediction of AKI [9,12,25,30]. In cases in which several limitations of biomarkers are present, the most beneficial biomarkers should be selected and subsequent studies should be performed to identify the applicable cutoff values in the clinical setting rather than in simple subgroups.

## 5. Conclusions

The 2012 KDIGO guidelines for AKI diagnosis remain the leading authority in real clinical settings, owing to their simplicity and effectiveness being validated in hundreds of studies. The standard methods for AKI assessment and treatment include selecting high-risk patients, measuring biomarkers for early detection, optimizing volume status, and reviewing medications. While some centers and studies have maintained a “wait-and-see” strategy, others have attempted to apply emerging diagnostic tools and protocols. The current biomarkers reported in the literature have several limitations that need to be overcome. Further studies are needed to reach a consensus on biomarker-guided management of AKI. A clearer stratification of high-risk cases and AKI sub-phenotypes and the integration of appropriate biomarkers are needed to advance routine clinical practice.

## Figures and Tables

**Table 1:** Biomarkers of AKI (adapted from [7,8]).

AKI Biomarker	Biological Role (Source)	Type of Marker (Sample)	Time of Increase after Injury	Limitations (Studied Population)
Alanine aminopeptidase; alkaline phosphatase; γ-glutamyl transpeptidase	Located in proximal tubular cells; released into urine after tubular damage ([9])	Damage (urine)		Elevated in UTI, cardiovascular disease, and stroke (patients in the ICU)
Cystatin C	Produced by nucleated human cells; freely filtered ([8,9,10])	Functional (plasma)	12–24 h after injury	Confounded by age, sex, inflammatory state, diabetes, low albumin level, muscle mass, and use of high-dose steroids (patients undergoing cardiac surgery or liver transplantation; hospitalized patients)
Hepcidin	Predominantly produced in hepatocytes; freely filtered ([10])	Damage (urine and plasma)		Decreased in anemia and increased in an inflammatory state (patients undergoing cardiac surgery; patients in the ICU)
Tissue metalloproteinase-2; insulin-like growth factor binding protein-7	Metalloproteinases released during cell-cycle arrest ([8,12,25])	Stress (urine)	As early as 4 h but typically within 12 h	Elevated in diabetes (patients undergoing cardiac or noncardiac surgery; patients in the ICU; patients in the ED)
Interleukin-18	Released into urine after tubular damage ([9,10])	Damage (urine)		Elevated in an inflammatory state; lack of cutoff values (hospitalized patients; patients in the ICU or ED; patients undergoing cardiac surgery)
Kidney injury molecule-1	Produced by proximal tubular cells; released into urine after tubular damage ([8,9,10])	Damage (urine)	12–24 h after injury	Elevated in chronic proteinuria and inflammatory diseases (hospitalized patients; patients in the ED; patients undergoing cardiac surgery; patients in the ICU)
Liver-type fatty acid-binding protein	Freely filtered and reabsorbed in proximal tubules; released into urine after tubular cell damage ([10])	Damage (urine and plasma)		Associated with anemia in patients without diabetes (patients undergoing cardiac surgery; patients in the ICU or ED)
N-acetyl-β-D-glucosaminidase	Released into urine after tubular damage ([8,11])	Damage (urine)	Within 2–4 h after injury	Elevated in diabetes and albuminuria (patients undergoing cardiac surgery; hospitalized patients)
Neutrophil gelatinase-associated lipocalin	At least three different types: (1) produced by neutrophils and epithelial tissues, including tubular cells; (2) produced by neutrophils; and (3) produced by tubular cells ([9,10,11])	Damage (urine and plasma)		Elevated in sepsis, UTI, and CKD; lack of specific cutoff values (patients undergoing cardiac or noncardiac surgery; patients undergoing coronary angiography; patients in the ICU; post-transplantation patients; patients in the ED)
Proenkephalin A	Freely filtered ([26])	Functional (plasma)		(Patients in the ICU; patients undergoing cardiac surgery; hospitalized patients)

AKI, acute kidney injury; CKD, chronic kidney disease; ED, emergency department; ICU, intensive care unit; UTI, urinary tract infection.

**Table 2:** Biomarkers of AKI progression and reversal (adapted from [7,8]).

AKI Biomarker	Biological Site (Source)	Type of Marker (Sample)	Time of Increase after Injury	Limitations (Studied Population)
C-C motif chemokine ligand 14	Released into urine after stress or damage to tubular cells ([8,13])	Damage (urine)	To identify patients who will develop persistent AKI for >72 h	Variable performance in different AKI phenotypes (patients in the ICU)
Hepatocyte growth factor	Produced by mesenchymal cells and involved in tubular cell regeneration after AKI ([14])	Damage (plasma)		Limited performance (hospitalized patients)
Monocyte chemoattractant peptide-1	Expressed in tubular epithelial cells, kidney mesangial cells, and podocytes ([15])	Damage (urine)		(Patients undergoing cardiac surgery)

AKI, acute kidney injury; ICU, intensive care unit.

## Data Availability

Not applicable.
